# Supplementary material for: Mechanisms governing the pioneering and redistribution capabilities of the non-classical pioneer PU.1
Source: Nat Commun. 2020 Jan 21;11:402. doi: 10.1038/s41467-019-13960-2 (PMC6972792; doi:10.1038/s41467-019-13960-2)
Supplement: Supplementary file 7 — Source data [file 41467_2019_13960_MOESM7_ESM.zip › Source_Data/FigureS2/FigureS2B_MotifScanOutput/KmeansCluster6/homerResults/motif14.similar.html]

motif14

## Information for motif14

A
T
G
C
A
G
T
C
A
C
G
T
A
G
C
T
A
G
T
C
G
A
T
C
C
T
G
A
G
A
T
C
A
G
T
C
C
G
T
A
A
C
G
T
C
T
A
G
  
Reverse Opposite:  

G
A
T
C
T
G
C
A
A
C
G
T
C
T
A
G
C
T
A
G
G
A
C
T
C
T
A
G
C
T
A
G
T
C
G
A
C
G
T
A
C
T
A
G
T
A
C
G
  

|  |  |
| --- | --- |
| p-value: | 1e-62 |
| log p-value: | -1.441e+02 |
| Information Content per bp: | 1.712 |
| Number of Target Sequences with motif | 206.0 |
| Percentage of Target Sequences with motif | 6.26% |
| Number of Background Sequences with motif | 572.8 |
| Percentage of Background Sequences with motif | 1.33% |
| Average Position of motif in Targets | 97.0 +/- 65.2bp |
| Average Position of motif in Background | 103.1 +/- 68.5bp |
| Strand Bias (log2 ratio + to - strand density) | -0.3 |
| Multiplicity (# of sites on avg that occur together) | 1.18 |
| Motif File: | file (matrix) reverse opposite |

### Similar de novo motifs found

|  |  |  |  |  |  |  |  |
| --- | --- | --- | --- | --- | --- | --- | --- |
| Rank | Match Score | Redundant Motif | P-value | log P-value | % of Targets | % of Background | Motif file |
| 1 | 0.790 | A G T C G T A C A C G T T A C G C T A G C G A T C T A G C T A G C T A G G T C A C T A G C T A G G A C T C T A G | 1e-40 | -92.794736 | 1.91% | 0.13% | motif file (matrix) |
| 2 | 0.772 | A G T C G A T C T C G A A G T C G A T C C T G A A G C T C T A G C T G A | 1e-33 | -77.600297 | 5.38% | 1.72% | motif file (matrix) |
